# Expressional divergence of the fatty acid-amino acid conjugate-hydrolyzing aminoacylase 1 (L-ACY-1) in *Helicoverpa armigera* and *Helicoverpa assulta*

**DOI:** 10.1038/s41598-017-09185-2

**Published:** 2017-08-18

**Authors:** Qian Cheng, Shaohua Gu, Zewen Liu, Chen-Zhu Wang, Xianchun Li

**Affiliations:** 1 Key Laboratory of Integrated Management of Crop Diseases and Pests (Ministry of Education), College of Plant Protection, Nanjing Agricultural University, Nanjing, 210095 China; 2 State Key Laboratory for Biology of Plant Diseases and Insect Pests, Institute of Plant Protection, Chinese Academy of Agricultural Sciences, Beijing, 100193 China; 3 State Key Laboratory of Integrated Management of Pest Insects and Rodents, Institute of Zoology, the Chinese Academy of Sciences, Beijing, 100101 China; 4 Department of Entomology and BIO5 Institute, The University of Arizona, Tucson, 85721 AZ USA

## Abstract

How FACs-producing generalist and specialist herbivores regulate their FACs-hydrolyzing enzyme L-ACY-1 to balance FACs’ beneficial vs. detrimental effects remains unknown. To address this question, we compared *L-ACY-1* expression in *Helicoverpa armigera* and *Helicoverpa assulta*, a pair of closely related sibling species differing mainly in their host range, by the same sets of hostplants, protein to digestible carbohydrate (P:C) ratios, or allelochemical. *L-ACY-1* expression remained low/unchanged in *H. armigera*, but was induced by hot pepper fruits and repressed by cotton bolls in *H. assulta*. The representative allelochemicals of the tested hostplants significantly (capsaicin) or insignificantly (gossypol and nicotine) induced *L-ACY-1* expression in *H. armigera*, but insignificantly inhibited (capsaicin and gossypol) or induced (nicotine) it in *H. assulta. L-ACY-1* expression remained low/unaltered on balanced (P50:C50 and P53:C47) or protein-biased diets and induced on carbohydrate-biased diets in *H. armigera*, but was at the highest level on balanced diets and reduced on either protein- or carbohydrate-biased diets in *H. assulta*. Furthermore, *L-ACY-1* expression was significantly higher in *H. assulta* than in *H. armigera* for most of feeding treatments. Such expressional divergences suggest that FACs are utilized mainly for removal of excessive nitrogen in generalists but for nitrogen assimilation in specialists.

## Introduction

While plants cannot flee from attacking insect herbivores, they often exploit certain chemical components of insect oral secretions and/or oviposition fluids as elicitors to mount their direct (e.g. anti-herbivore allelochemicals and proteinase inhibitors) and indirect defenses [natural enemies-attracting volatile organic compounds (VOCs)]^1–5^. Volicitin, first identified from the oral secretion of beet armyworm (*Spodoptera exigua*) larvae^6^, and the related glutamine- or glutamate-based fatty acid-amino acid conjugates (FACs) are among the most broadly-recognized plant defense elicitors; plants in Poaceae (maize), Fabaceae (soybean), Solanaceae (eggplant) and possibly additional families can recognize them^7^. Nonetheless, *Drosophila melanogaster*, two cricket species (*Teleogryllus taiwanemma* and *T. emma*) and more than two-thirds of lepidopteran species so far tested incorporate their glutamine with plant-derived fatty acids [mostly linoleic (18:2) and linolenic (18:3) acids] into FACs in the midgut and release part of the synthesized FACs into their oral secretions or regurgitates^5, 8–11^. Apparently, these insects must gain benefits from FACs that are worth the cost of triggering direct and indirect defenses in their hostplants. A recent study of ammonia assimilation, FACs biosynthesis, and glutamine uptake in *Spodoptera litura* larvae demonstrates that FACs play an essential role in nitrogen assimilation by functioning not only as a sink for glutamine through depleting glutamine in midgut cells but also as a primary storage of glutamine synthesized from glutamic acid and ammonia by glutamine synthetase (GS)^12^.

Given the aforementioned conflicting effects of FACs, it is essential for the FACs-producing insect herbivores to regulate the amount of FACs in their gut lumens and oral secretions. The finding of different abundance of FACs hydrolysis enzyme in the oral secretions and gut lumens as the major factor affecting the FACs amount in the oral secretions and frass of the closely-related *Heliothis virescens* and *Helicoverpa zea* ^13^ suggests that FACs-producing insects rely on decomposition of FACs to adjust the amount of FACs. The enzyme responsible for the hydrolysis of FACs to fatty acids and amino acids has recently been identified as a Lepidopteran aminoacylase 1 (L-ACY-1) in *H. virescens* and the gene has been cloned/sequenced in *H. zea*, *H. armigera* and *Spodoptera frugiperda* ^14^.

Aminoacylase 1 (ACY 1) is a cytosolic, homodimeric, zinc-binding enzyme that is also expressed primarily in kidney, liver and intestine^15, 16^ of mammals to catalyze the hydrolysis of N-acyl-L-amino acids to L-amino acids and acyl group for the catabolism and salvage of acylated amino acids^17–19^. Consistent with the greater batch-wise variability of FACs in the oral secretion of *H. virescens* than in that of *H. zea* and the larger amount of undegraded FACs in the frass of *H. zea* than in that of *H. virescens* ^13^, L-ACY-1 transcript level, protein abundance and activity in gut tissue and frass are the highest in *Heliothis subflexa*, a *Physalis* specialist, followed by *H. virescens*, a polyphagous Lepidopteran, and the least in *H. zea* ^20^, one of the most polyphagous Lepidopteran with a broader hostplant range than *H. virescens* ^21^. Such an inverse correlation among FACs stability and level in oral secretion and frass, L-ACY-1 abundance, and hostplant range implies that insects with different hostplant range regulate FACs amount differentially. Because FACs and L-ACY-1 are involved in nitrogen assimilation^12^, differential L-ACY-1 abundance and thus differential regulation of FACs in insects with different hostplant range are probably due to their different ability to obtain nutrients and different protein intake optimums^20^.

The above inverse relationship among L-ACY-1 abundance, FACs level and hostplant range was revealed from larvae of *H. subflexa*, *H. virescens*, and *H. zea* maintained on a common artificial diet^20^. It remains unclear whether this inverse correlation between L-ACY-1 abundance and hostplant range holds true when other specialist and generalist herbivores are compared and when they are fed with tissues or organs of host plants. Furthermore, attribution of the inverse relationship to the different ability to obtain nutrients and different intake optimums of the specialist and generalist insects^20^ is a conceivable explanation, but it has not been experimentally tested yet.

FACs’ ability to elicit plant defenses (e.g. production of VOCs and allelochemicals)^6, 7^ and L-ACY-1’s function to break down the plant defense elicitor FACs^14^ clearly show that L-ACY-1 is not only involved in insects’ nitrogen assimilation, but also acts as an insect counter-defense gene to minimize elicitation of plant defenses by decomposition of FACs. Insect counter-defense genes, such as allelochemicals-metabolizing Cytochrome P450 monooxygenases (P450s), glutathione S-transferases (GSTs) and esterases, are often upregulated by plant defense end product allelochemicals^2, 22^. Thus, it is logical to hypothesize that the insect counter-defense gene *L-ACY-1* may also be differentially regulated by allelochemicals in specialist and generalist herbivores, which in turn may contribute, at least partially, to the relationship between L-ACY-1 abundance and diet breadth.

*H. armigera* and *H. assulta* are a pair of closely related hybridizable sympatric sibling species differing mainly in their hostplant range^21, 23–26^. On the one hand, *H. armigera* is one of the most polyphagous species, with a host range consisting of at least 60 crop species and 67 wild plant species from about 30 different plant families, including Malvaceae, Leguminosae, Gramineae and Solanaceae^25, 27^. On the contrary, *H. assulta* feeds exclusively on several plants such as tobacco and hot pepper in Solanaceae^25, 27^. In this study, we took the opportunity offered by the two *Helicoverpa* species to address the above three general questions. We subjected the newly-molted final instar larvae of both species to the same sets of hostplant, protein to digestible carbohydrate (P:C) ratio or allelochemical treatments for 48 h and then used qRT-PCR to quantify the expressional differences of *L-ACY-1* in the midguts of the two sister species. We found that *L-ACY-1* expression was differentially regulated by allelochemicals, P:C ratios and hostplants in the two sibling species, and was significantly higher in the specialist *H. assulta* than the generalist *H. armigera* for most of the feeding treatments. These data suggest that FACs are utilized mainly for removal of excessive nitrogen in generalists but for nitrogen assimilation in specialists.

## Results

### *L-ACY-1* cDNA in *H.assulta*

**Figure 1:**
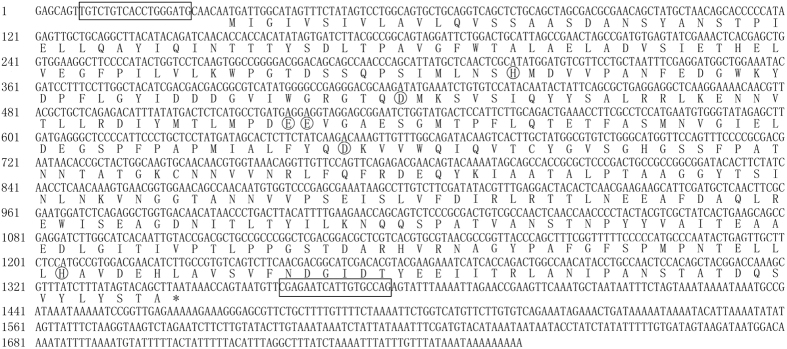
The full length cDNA sequences of *H. assulta* *L-ACY-1*. ○ = zinc-binding site of L-ACY-1.

**Figure 2:**
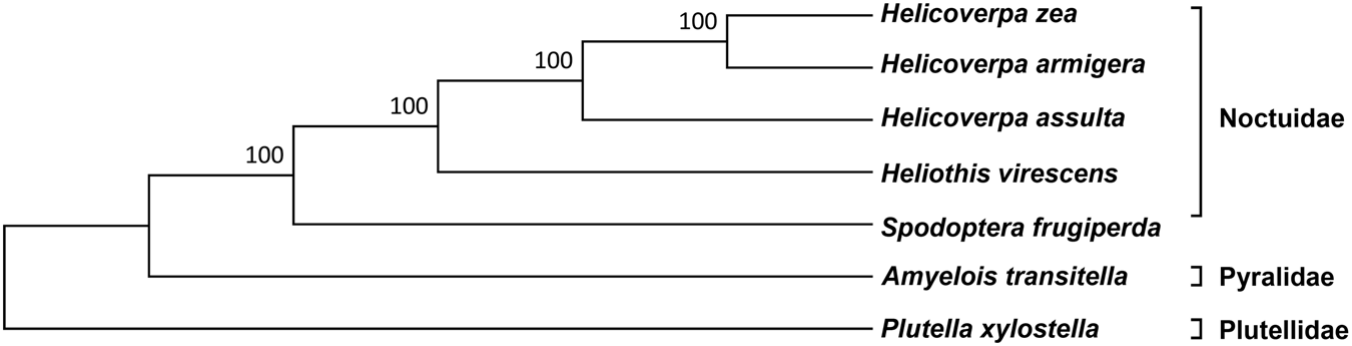
Phylogenetic tree of insect L-ACY-1 based on their amino acid sequences. Phylogenetic tree was constructed using the neighbor-joining method with 1000 bootstrap replicates. The numbers at each tree node are the bootstrap values.

The full-length cDNA sequence of *L-ACY-1* from *H. assulta* (NCBI accession number KY348705) was obtained by RT-PCR cloning of its middle fragment (1199 bp) with a pair of primers (Table 1) designed based on *H. armigera L-ACY-1*, followed by cloning of its 5′ and 3′ ends by 5′ and 3′ RACE (random amplification of cDNA ends), and cloning of its full-length sequence with the two full-length primers (Table 1) designed based on its 5′ and 3′ ends. The *H. assulta L-ACY-1* is 1758 bp long, containing a 30 bp 5′ UTR (5′ untranslated region), an ORF (open reading frame) of 1314 bp (from 31 bp to 1344 bp) encoding 437 amino acids, and a 414 bp 3′ UTR (Fig. 1). The deduced *H. assulta* L-ACY-1 protein shares 95% amino acid identity with *H. armigera* L-ACY-1, 96% with *H. zea* L-ACY-1, 88% with *H. virescens* L-ACY-1, 69% with *S. frugiperda* L-ACY-1, 56% with *Plutella xylostella* L-ACY-1, 63% with *Amyelois transitella* L-ACY-1. Phylogenetic analysis reveals that *H. assulta* L-ACY-1 belongs to the Noctuidae lineage and is the immediate common ancestor of *H. armigera* and *H. zea* L-ACY-1 (Fig. 2), consistent with the evolutionary relationship among the 3 *Helicoverpa* species^23, 28, 29^. Like other aminoacylases, *H. assulta* L-ACY-1 has a dinuclear zinc-binding site, which is composed of His96 Asp130, Glu164 Glu165 Asp207 and His392 (circled amino acids in Fig. 1).

**Table 1:** Primer pairs used for cloning of *H. assulta* L-ACY-1.

Gene name (abbreviation)	Sequence (5′-3′)^a^	Product length (bp)
*H. ass*-L-ACY-1-partial cloning	F:CCAACAGCACCCCCATAG	1199
	R: GGAGTTAGCAGGTAAGTTGGC	
*H. ass*-ACY-3′	ATCGGCTAGTTCGGCTAATGCGGTCC	
5′-RACE CDS Primer A	5′-AAGCAGTGGTATCAACGCAGAGTAC(T) _25_V N-3′	
Clontech SMARTer II A Oligonucleotide	5′-AAGCAGTGGTATCAACGCAGAGTACATGGG-3′	
*H. ass*-ACY-5′	CTTTCGGTTTTTCCCCCATGCCCAAT	
3′-RACE CDS Primer A	5′-AAGCAGTGGTATCAACGCAGAGTAC(T)30 V N-3′	
UPM, long	5′-CTAATACGACTCACTATAGGGCAAGC AGTGGTATCAACGCAGAGT-3′	
UPM, short	5′-CTAATACGACTCACTATAGGGC-3′	
*H. ass*-L-ACY-1- full-length cloning	F: TGTCTGTCACCTGGGATG	1370
	R: CTGGCACAATGATTCTCG	

^a^F and R respectively indicate forward primer and reverse primer.

### Impacts of hostplants on *L-ACY-*1 expression in *H. armigera* and *H. assulta*

**Figure 3:**
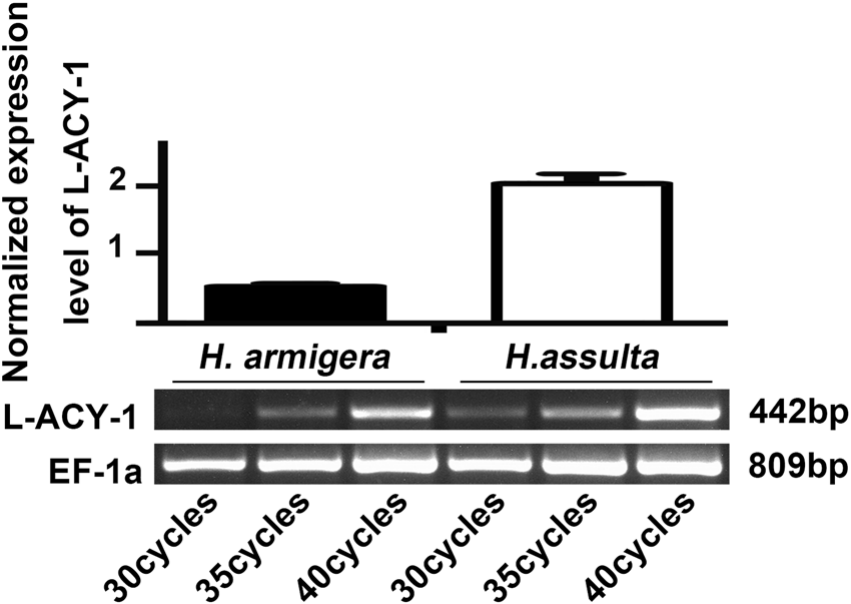
Transcriptional levels of *L-ACY-1* in the larval midguts of *H. armigera* and *H. assulta* maintained on the control diet. Means and their standard deviations shown in the chart are obtained from qRT-PCR analysis of at least three biological replicates of three technical repeats each for each species. Significant difference between the two species is indicated by one star (*p < 0.05, independent t-test). Below the chart is a representative gel picture of semi-quantitative RT-PCR analysis of *L-ACY-1* and *EF-1a* (reference gene) transcripts.

**Figure 4:**
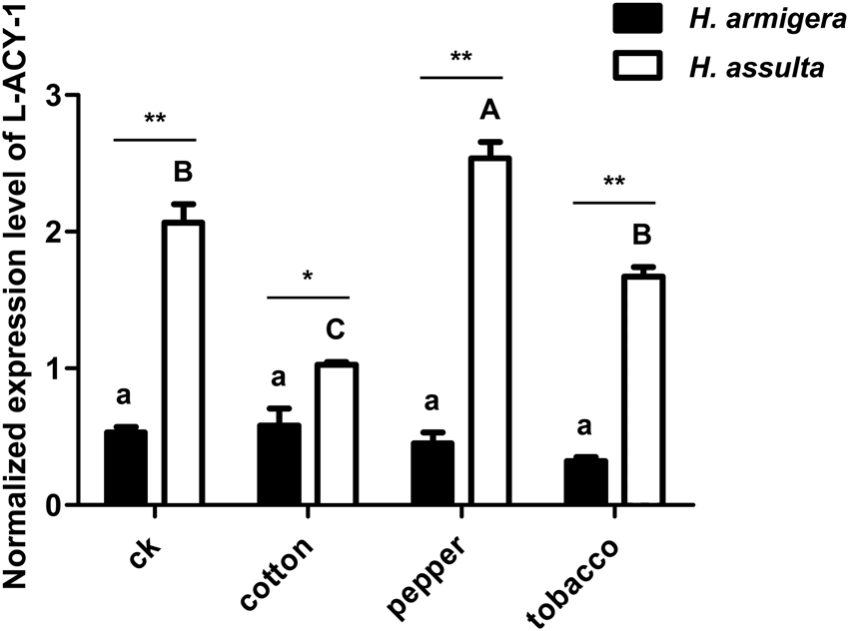
Effects of hostplants on the expression of *L-ACY-1* in *H. armigera* and *H. assulta*. The data and error bars represent the means and standard deviations of three biological replicates of three technical repeats each. Values sharing the same letter [small case letters for *H. armigera*, capital letters for *H. assulta*] are not significantly different at *p* < 0.05 (Tukey’s HSD tests). Significant differences at each of the four treatments between the two species are indicated by one star (*p < 0.05, independent t-test) and two stars (**p < 0.01, independent t-test), respectively.

RT-PCR gel analysis showed that when the larvae of both species were reared on the same wheat germ-containing artificial diets [i.e. the control diet, 53 protein (P): 47 carbohydrate (C)]^30^, *L-ACY-1* transcripts were about 3.89 fold higher in the specialist *H. assulta* than its generalist counterpart *H. armigera* (Fig. 3), consistent with the qRT-PCR data shown in Fig. 4 (see control). When the newly-molted final instar larvae of both species were transferred onto detached tobacco leaves (common host plants for both species), cotton bolls (cut in half; favored host plant for *H. armigera* but poor for *H. assulta*), or hot pepper fruits (favored host plant for *H. assulta* but poor for *H. armigera*) and reared for 48 h, *L-ACY-1* expression remained unchanged relative to the larvae fed on the control diets in *H. armigera* (Fig. 4). By contrast, *L-ACY-1* expression in *H. assulta* was very sensitive to hostplant species; cotton bolls significantly repressed *L-ACY-1* expression, whereas pepper fruits significantly induced it (Fig. 4); tobacco leaves did not cause significant changes in *L-ACY- 1* expression. Cross-species comparison revealed that *L-ACY-1* expression was significantly higher in *H. assulta* than in *H. armigera* in both the control diets and the three tested hostplants, with a *H. assulta* / *H. armigera* ratio ranged from 1.77 (cotton bolls) to 5.21 (tobacco leaves), 3.90 (control diets) or 5.62 fold (hot pepper fruits) (Fig. 4).

### Effects of plant allelochemicals on *L-ACY-1* expression in *H. armigera* and *H. assulta*

**Figure 5:**
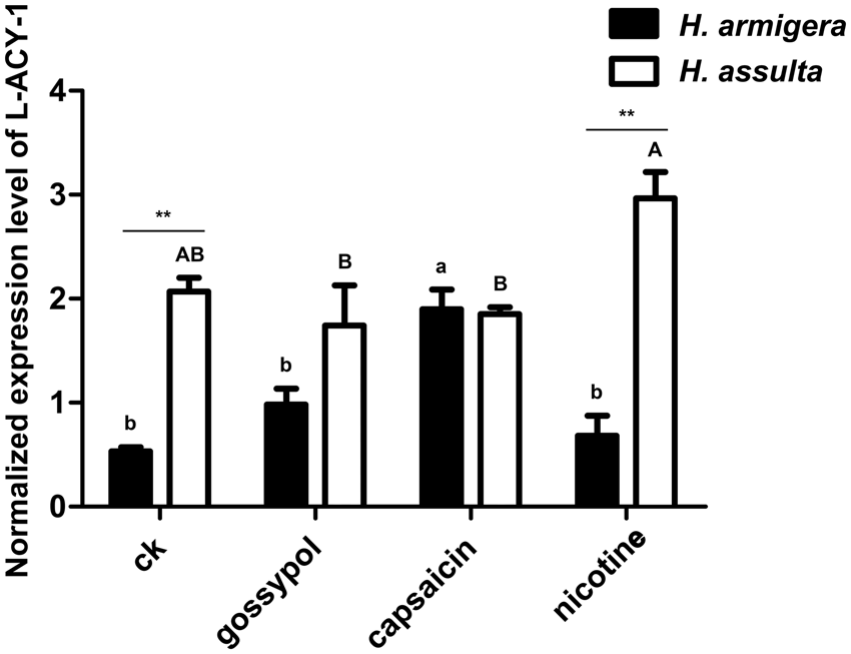
Effects of allelochemicals on the expression of *L-ACY-1* in *H. armigera* and *H. assulta*. The data and error bars represent the means and standard deviations of three biological replicates of three technical repeats each. Values sharing the same letter [small case letters for *H. armigera*, capital letters for *H. assulta*] are not significantly different at *p* < 0.05 (Tukey’s HSD tests). Significant differences at each of the four treatments between the two species are indicated by one star (*p < 0.05, independent t-test) and two stars (**p < 0.01, independent t-test), respectively.

To determine whether allelochemicals present in their common (capsaicin in hot pepper and nicotine in tobacco) or unique (gossypol in cotton for *H. armigera*) natural hostplants account for their differential regulation of *L-ACY-1* expression in response to the three hostplants, we subjected the newly-molted final instar larvae of both species to control diets or diets supplemented with 0.1% capsaicin, nicotine, or gossypol for 48 h and then analyzed the expression of *L-ACY-1* in the midguts of these larvae by qRT-PCR. Two-way ANOVA showed significant impacts of insect species (Df = 1, F = 61.7, P = 0.00), allelochemicals (Df = 3, F = 4.4, P = 0.02) and their interactions (Df = 3, F = 12.1, P = 0.00) on *L-ACY-1* expression. In *H. armigera*, capsaicin (3.58-flod) significantly induced the expression of *L-ACY-1*, gossypol (1.85-flod) and nicotine (1.28 fold) slightly but not significantly increased the expression of *L-ACY-1* (Fig. 5). In *H. assulta*, nicotine (1.44-flod), slightly induced the expression of *L-ACY-1*, whereas capsaicin (0.90-flod) and gossypol (0.84-flod) slightly inhibited the expression of *L-ACY-1* (Fig. 5). Cross-species comparison revealed that *L-ACY-1* expression was significantly higher in *H. assulta* than in *H. armigera* when their larvae were fed on control diets (3.90 fold) or nicotine-containing diets (4.35-flod). When their larvae were fed on diets supplemented with capsaicin (*p* = 0.823) or gossypol (*p* = 0.141), the expression of *L-ACY-1* in *H. assulta* was similar to or insignificantly higher than that in *H. armigera* (Fig. 5).

### Effects of protein to digestible carbohydrate ratios on *L-ACY-*1 expression in *H. armigera* and *H. assulta*

**Figure 6:**
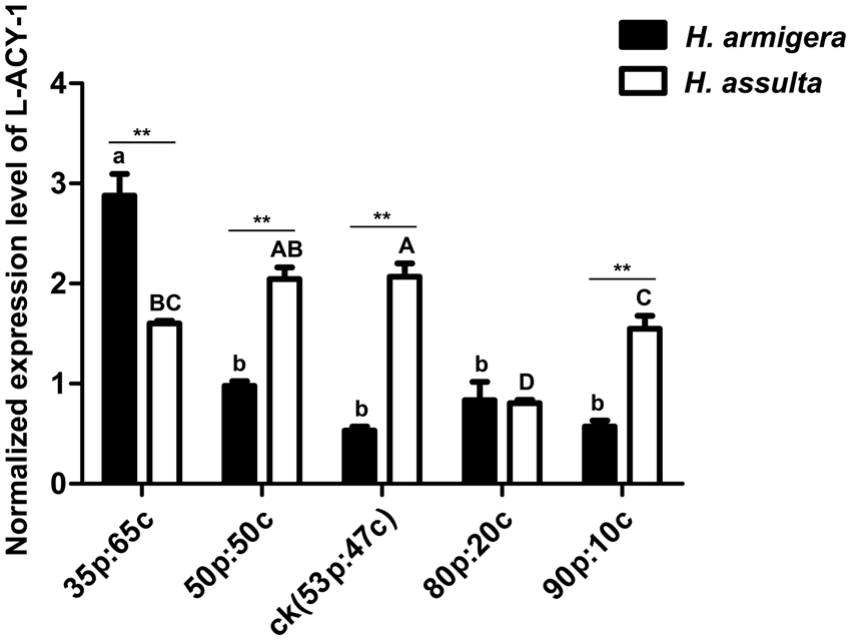
Effects of protein to digestible carbohydrate (P:C) ratios on the expression of *L-ACY-1* in *H. armigera* and *H. assulta*. The data and error bars represent the means and standard deviations of three biological replicates of three technical repeats each. Values sharing the same letter [small case letters for *H. armigera*, capital letters for *H. assulta*] are not significantly different at *p* < 0.05 (Tukey’s HSD tests). Significant differences at each of the five treatments between the two species are indicated by one star (*p < 0.05, independent t-test) and two stars (**p < 0.01, independent t-test), respectively.

To determine if protein to digestible carbohydrate (P:C) ratio variable in their natural host plants affects *L-ACY-1* expression in the two sibling species, we fed the newly-molted final instar larvae of both species on artificial diets with a P:C ratio of 35:65, 50:50, 53:47 (control diets for rearing the lab strains of both species), 80:20, or 90:10 for 48 h and then quantified the expression of *L-ACY-1* in the midguts of these larvae by qRT-PCR. Two-way ANOVA uncovered significant effects of insect species (Df = 1, F = 37.8.7, P = 0.00), P:C ratios (Df = 4, F = 43.1, P = 0.00) and their interactions (Df = 4, F = 46.2, P = 0.00) on *L-ACY-1* expression. In *H. assulta*, the larvae feeding on the balanced 50 P:50 C diets and near-balanced 53 P:47 C control diets had the highest level of *L-ACY-1* expression, whereas the larvae feeding on either lower (35:65) or higher (80:20 and 90:10) P:C ratio diets exhibited significantly reduced expression of *L-ACY-1* (Fig. 6). By contrast, *H. armigera* larvae feeding on the balanced (50 P:50 C) and higher P:C ratio diets (53:47, 80:20 and 90:10) had the similar level of *L-ACY-1* expression; reducing the P:C ratio to 35:65 significantly induced *L-ACY-1* expression (Fig. 6). Between-species comparison found significantly higher expression of *L-ACY-1* in *H. assulta* than *H. armigera* when their larvae were fed on the 50:50, 53:47 or 90:10 P:C ratio diets, but significantly lower expression on the 35:65 P:C ratio diets. No significant between-species difference in *L-ACY-1* expression was observed on the 80:20 diets (Fig. 6).

## Discussion

FACs play a beneficial role in nitrogen assimilation^12^ of lepidopteran herbivores while harming them by eliciting direct and indirect plant defenses^1–5^. Excretion of FACs into the oral secretions and frass of these herbivores^13^ indicate that FACs also help them to remove overeaten nitrogen, which is toxic to insects and thus must be excreted. Because generalist and specialist herbivores differ in their capacity to acquire macronutrients such as proteins and carbohydrates and to counteract plant defenses, they are expected to differentially regulate the expression of *L-ACY-1* to adjust FACs amount in their oral secretions and frass and thus balance the above three conflicting roles (nitrogen assimilation, nitrogen removal, and triggering plant defenses) of FACs.

To test this general postulation, we compared the expression profiles of the FACs-decomposing *L-ACY-1* in *H. armigera* and *H*. *assulta* final instar larvae fed with hostplant tissues, diets with different allelochemicals, or diets with different P:C ratios. Consistent with the reverse relationship between *L-ACY-1* expression and host range from studies of *H. virescens*, *H. subflexa* and *H. zea* maintained on a common artificial diet^13, 20^, *L-ACY-1* transcription was significantly greater in the specialist *H. assulta* than in the generalist *H. armigera* when they were fed on the common near-balanced control diet (Figs 3 and 4). Such a reverse relationship held true (Fig. 4) even we offered their larvae with tissues of cotton (bolls), a preferred hostplant for *H. armigera* but a non-host for *H. assulta*, tobacco (leaves), a hostplant commonly utilized by both species, and hot pepper (fruits), a common host for *H. assulta* but an occasional host for *H. armigera* ^25, 31–33^. That being said, the between-species *L-ACY-1* transcriptional difference (*H. assulta / H. armigera*) increased in the larvae fed with hot pepper fruits, but markedly reduced in those fed with cotton bolls, relative to that of the larvae on the control diet (Fig. 4). This host-dependent variation was largely resulted from significant induction and suppression of *L-ACY-1* transcription in the specialist *H. assulta* but not in the generalist *H. armigera* by hot pepper fruits and cotton bolls, respectively.

Plant tissues such as tobacco leaves, hot pepper fruits and cotton bolls used in this study are usually inferior to artificial diets because they are not only nutritionally less adequate and balanced than the later^34–37^, but also contain plant defense end products such as allelochemicals^2, 31, 38^. The plant-dependent significant induction or repression of *L-ACY-1* in *H. assulta* but lower and stable expression of *L-ACY-1* in *H. armigera* in response to the same set of plant tissues (Fig. 4) implies *L-ACY-1* is differentially regulated by nutrient imbalance and plant defense end products in the two species. In agreement with this inference, gossypol and nicotine, the two representative allelochemicals found in cotton and tobacco respectively^31, 39, 40^, the preferred or commonly-used host plants of the generalist *H. armigera*, insignificantly induced *L-ACY-1* expression in *H. armigera* but marginally repressed (gossypol) or elevated (nicotine) *L-ACY-1* expression in *H. assulta* (Fig. 5). Capsaicin, a representative allelochemical found in *H. armigera*’s poor host hot pepper, significantly induced *L-ACY-1* expression in *H. armigera* but marginally inhibited it in *H. assulta*. The aforementioned species-specific opposite effects of capsaicin and gossypol rendered *L-ACY-1* expression level in *H. assulta* equal to that in *H. armigera*.

Overall, *L-ACY-1* transcription was more sensitive to allelochemicals in *H. armigera* than in *H. assulta*. The insignificant (gossypol and nicotine) or significant (capsaicin) induction of *L-ACY-1* in the generalist *H. armigera* is an intuitively expected self-protection response by reducing the FACs amount in its oral secretion and frass and thus minimizing elicitation of plant defenses. Induction of *L-ACY-1* may also increase use of FACs for nitrogen assimilation and thus protect this species via reduced allelochemical exposure by eating less food. By contrast, the lack of *L-ACY-1* induction in the specialist *H. assulta* by capsaicin is probably because *H. assulta* uses capsaicin as a feeding stimulant^41^ and is much more tolerant to capsaicin than *H. armigera* ^42^. That *H. assulta* does not naturally encounter gossypol in its restricted range of hostplants may explain why this allelochemical failed to induce *L-ACY-1* expression in this specialist. The overall lower sensitivity of *H. assulta L-ACY-1* expression to allelochemicals is probably because specialist detoxification enzymes are usually less inducible by allelochemicals and degrade the allelochemicals present in the hostplants of a given specialist at a much higher efficiency than do the corresponding generalist homologs^22, 43^.

Contrary to the allelochemical induction profiles of *L-ACY-1* in the two *Helicoverpa* species, *L-ACY-1* transcription was overall more sensitive to macronutrient (protein and carbohydrates) imbalance in the specialist *H. assulta* than in the generalist *H. armigera* (Fig. 6). On the one hand, *L-ACY-1* expression level in *H. armigera* remained low when P:C ratio ranged from the relatively balanced (50:50 and 53:47) to high P:C ratio diets (80:20 and 90:10), and significantly increased only when undereating proteins on a low P:C (35:65) ratio diet (Fig. 6). On the other hand, *L-ACY-1* expression in *H. assulta* was at the highest level on the two balanced diets (50:50 and 53:47), but significantly repressed when overeating either low (35:65) or high (70:20 and 90:10) P:C diets (Fig. 6). The fact that the plant tissue-dependent expression patterns of *L-ACY-1* in the two species are more similar to those elicited by the P:C ratio diets—lower and less sensitive in *H. armigera* than *H. assulta*—suggests that dietary imbalance in the two major macronutrients contributes more to the cross-species difference in the plant tissue-elicited *L-ACY-1* expression than allelochemicals.

Our data also suggest that nutritional imbalance plays a more important role than do allelochemicals in determining the inverse relationship between host-range and *L-ACY-1* expression on artificial diets and hostplants. Although the self-selected balanced P:C ratio (intake target) of lepidopteran herbivores ranges from 0.8–1.6 (1.1 to 1.6 for generalists and 0.8 to 1.1 for specialists)^44, 45^, herbivores seldom encounter a plant species or tissue that is perfectly balanced nutritionally. Instead, they are frequently forced to overingest protein on a high-protein hostplant or carbohydrate on a high-carbohydrate plant tissue. Correlated with their host range difference, generalists are capable of overeating larger nutrient excesses (especially protein) than specialists since the former naturally experience a greater degree of nutritional heterogeneity than do specialists^46–48^. While overconsumed carbohydrates can be converted into lipids and stored inside the body, the nitrogen excesses from overingested proteins must be excreted because it is toxic to insect herbivores. Because of their expected and documented greater ability to overeat surplus quantities of excessive protein nutrients^46–48^, generalists may often utilize FACs for removal of excessive nitrogen, rather than nitrogen assimilation. By contrast, it is probably not easy for specialists to get enough protein nutrients from their narrow range of hostplants. Thus, specialists may use FACs mainly for nitrogen assimilation, which requires hydrolysis of FACs by L-ACY-1.

The divergences in the relative importance of the three conflicting functions (nitrogen assimilation, nitrogen removal, and eliciting plant defenses) of FACs and thus of L-ACY-1 in specialists and generalists may account for the inverse relationship between *L-ACY-1* expression level and host range^13, 20^ (Figs 3–5) and the differential non-linear expression patterns of *L-ACY-1* caused by P:C ratios (Fig. 6). For generalists such as *H. armigera*, which are readily to obtain enough or even surplus nutrients from a wider host range^46–48^ and have a more powerful detoxification system to overcome the diversity and unpredictability of plant defenses^22^, nitrogen removal is expected to be the most important function of FACs/L-ACY-1, followed by nitrogen assimilation, and FACs decomposition to avoid elicitation of plant defenses. The lower *L-ACY-1* expression of *H. armigera* larvae on relatively balanced (50:50 and 53:47) to high P:C ratio diets (80:20 and 90:10) (Fig. 6) allows this species to excrete at least part of the overingested nitrogen in the form of FACs in its frass and oral secretions^13^. The significantly enhanced *L-ACY-1* expression when undereating proteins on a low P:C (35:65) diet (Fig. 6), on the other hand, increases this species’ ability to assimilate nitrogen by breaking down FACs into amino acids and lipids^12^.

By contrast, because of a narrower host range (thus greater difficulty to get enough nutrients) and a weaker detoxification system (thus less capacity to afford FACs elicitation of plant defenses), nitrogen assimilation becomes the most important function for specialists such as *H. assulta*, followed by FACs decomposition to avoid elicitation of plant defenses, and nitrogen removal. This explains why *L-ACY-1* expression remains at the highest level in the near-optimal 55:47 and 50:50 P:C diets, marginally reduce at the low P:C ratio diet (hydrolyzing FACs to avoid elicitation of plant defense and nitrogen assimilation), and significantly decrease at the high P:C ratio diets (nitrogen removal). Another possible reason for the marginal reduction of *L-ACY-1* expression in the low protein diet would be that plant tissues with lower P:C ratio often contain certain allelochemicals that are hard for specialists to degrade and thus *H. assulta* must lower *L-ACY-1* expression to avoid triggering production of the corresponding allelochemicals by plants. That cotton bolls, which have a P:C ratio of 0.41–1.17^45^ and contain gossypol, significantly repressed *L-ACY-1* expression in *H. assulta* but not in *H. armigera* (Fig. 4), supports this speculation.

## Materials and Methods

### Insect

The laboratory colonies of *H. armigera* and *H. assulta* used in this study were simultaneously established with newly-emerged F0 adults of each species developed from about 2100 larvae of *H. armigera* (about 60%) or *H. assulta* (about 40%) (indistinguishable at larval stage) collected on tobacco plants from Xvchang (Henan, China) in June 2015. The two species were then maintained in two separate insectaries kept at 28 °C with a photoperiod of 16 h light: 8 h dark, 70 ± 10% (for adults) or 40 ± 10% (for larvae) RH. Larvae of both species were reared separately and individually on wheat germ-containing artificial diets [53 protein (P): 47 carbohydrate (C)]^30^.

### Feeding experiments and preparation of midguts

Three different feeding experiments were conducted for both species with 3 biological replicates of 10 newly-molted final instar larvae each for all the treatments. In Experiment 1, we fed 30 newly-molted final instar larvae of each species with control diets, cotton bolls (cut in half; variety ‘Shiyuan 321’), leaves of 15–25 cm-tall tobacco plants (variety ‘Yabuli’) or fruits of hot pepper (variety ‘Xiaoxin 9’) collected from the corresponding plants grew in the open field in Langfang experimental station. In Experiment 2, we transferred 30 newly-molted larvae of each species from the control diet onto control diets or diets supplemented with 0.1% (W/W) of gossypol, capsaicin (Aimeida Company, Beijing) or nicotine (Sigma, USA). In Experiment 3, we transferred 30 newly-molted larvae of each species from the control diet (53 P:47 C) onto control diets or diets with a P:C ratio of 35:65, 50:50, 80:20 or 90:10. After 48 h of the feeding treatments mentioned above, we cooled the 30 larvae of each treatment on ice for about 10 mins, cut off the front and back ends of each larva between 2^nd^ and 3^rd^ thorax and immediately posterior to the last proleg segment, respectively, pulled out their midguts, open them to remove midgut contents, washed them in cold ddH_2_O, pooled them into 3 biological replicates of 10 midguts each, flash-frozen with liquid nitrogen, and stored in −80 °C for subsequent RNA extraction and analyses of *L-ACY-1* transcripts.

### RNA extraction and cDNA synthesis

Each replicate of 10 midguts were grinded to fine power in liquid nitrogen and transferred to a 1.5 ml centrifuge tube containing 600 µL of guanidinium-HCl RNA extraction buffer. Total RNA was extracted from the powder using the procedure described in Li *et al*.^49^, treated with DNase I (Promega, USA) and RNase inhibitor (Thermo, USA) for 40 min to remove potential genomic DNA (gDNA), purified by phenol/chloroform extraction and ethanol precipitation, and dissolved in 500–800 µL DEPC H_2_O. Total RNA samples were stored in −80 °C for subsequent RT-PCR cloning of *H. assulta L-ACY-1* gene and/or RT-PCR and qRT-PCR analyses of *L-ACY-1* expression.

### RT-PCR cloning and sequence analysis of *H. assulta L-ACY-1* gene

Cloning of the full-length cDNA sequence of *H. assulta L-ACY-1* began with RT-PCR amplification of its central region (1199 bp) with a pair of partial cloning primers (Table 1) designed based on *H. armigera L-ACY-1* (GenBank: JF922297.1), followed by 3′ rapid amplification of cDNA end (RACE) with the universal primer mix (UPM in the Clontech SMARTer^®^ RACE 5′/3′ Kit) and the gene-specific primer *H. ass*-ACY-5′ to amplify its 3′ end, 5′ RACE with UPM and the gene-specific primer *H. ass*-ACY-3′ (Table 1) to amplify its 5′ end, and amplification of its full-length cDNA with a pair of full-length cloning primers (Table 1). The initial partial PCR reaction (20 µL) was composed of 0.1 µL diluted *H. assulta* cDNA prepared above, 2 µL 10 × Ex Taq buffer, 0.1 µL Ex Taq (TaKaRa, Japan), 1.6 µL dNTP (2.5 nM), 200 pmol of the forward and reverse primer. The partial PCR reaction was initiated with 5 min denaturation at 95 °C, followed by 40 cycles of 30 s denaturation at 95 °C, 30 s annealing at 55 °C and 1.5 min extension at 72 °C, and a final 10 min extension at 72 °C on an Eppendorf Master Cycler.

Two µg of *H. assulta* total RNA, together with 1 µl 5′-RACE CDS Primer A and 1 µl SMARTer IIA oligo A or 1 µl 3′- RACE CDS Primer A were used to synthesize the 5′ or 3′ RACE-ready cDNAs with the SMART RACE cDNA Amplification kit (Clontech, USA) according to the manufacturer manual. The resultant 5′ or 3′ RACE-ready cDNA was used as the template to amplify the 5′ or 3′ end of *H. assulta L-ACY-1* with UPM and the gene-specific primer *H. ass*-ACY-5′ (5′ RACE) or *H. ass*-ACY-3′ (3′RACE) following the setup and touchdown cycling procedure described in the manufacturer manual.

The final full-length PCR reaction (50 µL) was composed of 3 µL diluted *H. assulta* cDNA, 5 µL 10 × LA Taq buffer, 0.25 µL LA Taq (TaKaRa, Japan), 0.03 µL Pfu (Agilent, USA), 1.6 µL dNTP (2.5 nM), 200 pmol of the forward and reverse primers. The cycling conditions were the same as for the initial partial PCR reaction described above. All the partial, 5′ RACE, 3′ RACE and full-length PCR products were run on 1.5% agarose gels, visualized by EB staining, gel-eluted with a Axygen kit, cloned into pGEM^®^-T Easy Vector (Promega, USA) and sequenced in BGI (Beijing, China).

The deduced amino acid sequences of *H. assulta* L-ACY-1 and other five lepidoptera insects were used for phylogenetic analysis. These orthologous proteins sequences were *H. armigera* (NCBI accession No.: AET43036.1), *H. zea* (AET43035.1), *H. virescens* (AET43034.1), *Plutella xylostella* (XP_011552700.1), *Spodoptera frugiperda* (AET43033.1), and *Amyelois transitella* (XP_013187226.1). The phylogenetic tree of L-ACY-1 was constructed with the MEGA 6 program, using the neighbor-joining method with 1000 bootstrap replicates.

### RT-PCR and qRT-PCR

**Figure 7:**
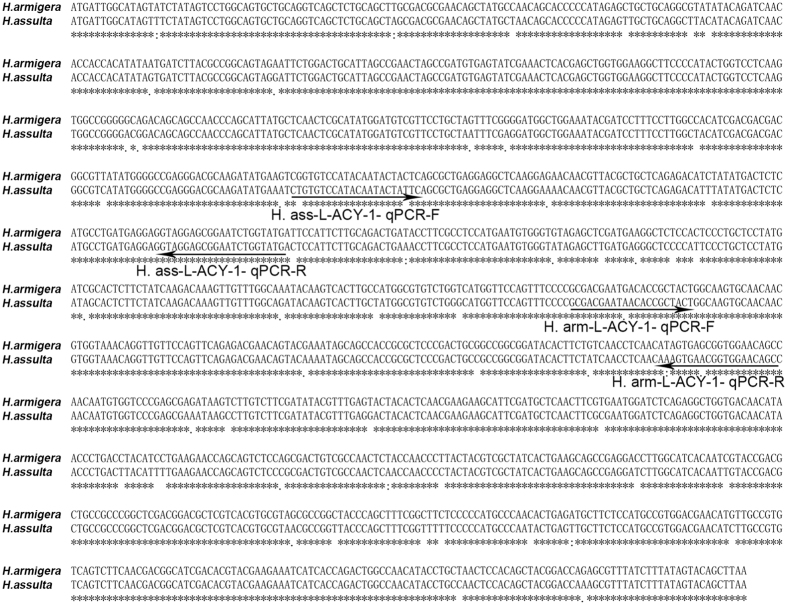
Nucleotide alignment of *H. armigera* and *H. assulta L-ACY-1* gene. The annealing positions of the two primer pairs used for qRT-PCR analysis of *L-ACY-1* in either species are depicted with arrowed lines and the corresponding primer names.

Two µg of each total RNA sample were reverse transcribed into cDNA at 42 °C for 1 h in a 20 µL reaction with 4 µL primer Mix (Tiangen, China), 4 µL dNTP (TaKaRa, Japan), 1 µL M-MuLV reverse transcriptase (New England Biolab) and 1.5 µL RNase inhibitor (Thermo, USA). The cDNAs were diluted 10-fold and used as the templates for RT-PCR gel analyses of *L-ACY-1* and *EF-1a* (as an internal reference gene) and qRT-PCR analyses of *L-ACY-1*, Ribosomal protein L-13 (*RPL-13*), ribosomal protein L-32 (*RPL-32*) and *β*-*Tubulin* in the two *Helicoverpa* species. The primer pairs for qRT-PCR analyses of the three reference genes (*RPL-13, RPL-32* and *β-Tubulin*) were from Songdou *et al*.^50^ (Table 2). The primer pairs for RT-PCR gel analyses of *L-ACY-1* and *EF-1a* (Table 3) and for qRT-PCR analyses of *L-ACY-1* (Fig. 7 and Table 2) were designed with Primer Premier 5. All the primers satisfy the standard of MIQE.

**Table 2:** Primer pairs used for qRT-PCR analysis of the target or reference genes. ^a^F and R respectively indicate forward primer and reverse primer. ^b^R^2^ refers to the coefficient of determination.

Gene name (abbreviation)	Sequence (5′-3′)^a^	Product length (bp)	Primer efficiency (%)	R^2 b^
*H. arm*-L-ACY-1- qPCR	F: GCGACGAATGACACCGCTACT	155	98.7571	0.9997
	R: GCTGTTCCACCGCTCACTATG			
*H. ass*-L-ACY-1- qPCR	F: CGGTGTCCATACAATACTACTC	119	92.1746	0.9901
	R: CATACCAGATTCCGCTCCTAC			
*H. arm*-RPL13 (Ribosomal protein L13)	F: CTGCAAGACGTCACCGCAG	139	91.799	0.9997
	R: CCACGACCAGCACGAACCT			
*H. ass*- RPL13 (Ribosomal protein L13)	the same with *H. arm* -RPL13		94.2788	0.9981
*H. arm*-RPL32 (Ribosomal protein L32)	F: CATCAATCGGATCGCTATG	152	94.48	0.9992
	R: CCATTGGGTAGCATGTGAC			
*H. ass*- RPL32 (Ribosomal protein L32)	the same with *H. arm* -RPL32		93.2776	0.9968
*H. arm*-β-TUB (beta-Tubulin)	F: AGCAGTTCACCGCTATGTTC	106	91.027	0.9998
	R:AGGTCGTTCATGTTGCTCTC			
*H. ass*-β-TUB (beta-Tubulin)	the same with *H. arm* -β-TUB		96.5231	0.9986

**Table 3:** Primer pairs used for RT-PCR analysis of the target or reference genes. ^a^F and R respectively indicate forward primer and reverse primer.

Gene name (abbreviation)	Sequence (5′-3′)^a^	Product length (bp)
L-ACY-1- RT-PCR	F: CCAACAGCACCCCCATAG	442
	R: ATACCAGATTCCGCTCCTACC	
EF-la- RT-PCR (Elongation factor 1 alpha)	F: GACAAACGTACCATCGAGAAG	809
	R: GGTACAGCCTCCTGGAGAGC

RT-PCRs of *H. armigera* and *H. assulta L-ACY-1* and *EF-1a* were conducted individually in a 20 μL reaction composed of 1 µL 10 fold-diluted cDNA, 2 µL 10 × *Ex Taq* buffer, 0.1 µL *Ex Taq* (TaKaRa, Japan), 1.6 µL dNTP (2.5 nM), and 400 pmol *L-ACY-1*-specific primer pair (Table 3; 200 pmol each) or *EF-1a*-specific primer pair (Table 3; 20 pmol each). The common PCR cycling conditions included an initial denaturation at 95 °C for 5 min, followed by 30, 35, or 40 cycles of denaturation at 95 °C for 30 s, annealing at 55 °C (*L-ACY-1*) or 60 °C (*EF-1a*) for 30 s and extension at 72 °C for 1 min, and a final extension at 72 °C for 5 min. The resultant RT-PCR products (20 µL) were fractioned on a 1.5% agarose gel containing 1 × TAE buffer, visualized by EB staining and quantified with ImageJ.

qPCRs of *H. armigera* and *H. assulta L-ACY-1, RPL-13, RPL-32* and *β*-*Tubulin* were individually performed in a 20 µL reactions containing 1 µL 10 fold-diluted cDNA, 200 pmol gene-specific primer pair (Table 2; 100 pmol each), 10 µL 2X GoTaq^®^ qPCR Master Mix and 0.4 µL CXR Reference Dye (50×) (Promega, USA) using an ABI 7500 Real-Time PCR System (Applied Biosystems, Foster City, CA). The cycling program was consisted of an initial denaturation at 95 °C for 15 min, followed by 40 cycles of denaturation at 95 °C for 15 s, annealing at 60 °C for 30 s and extension at 72 °C for 32 s, during which data collection and real-time analysis enabled. Melting curve analysis was performed from 65 °C to 95 °C for *L-ACY-1* and the three reference genes to ensure no interference of junk products. Each biological replicate was qRT-PCR-analyzed in triplicate. Amplification efficiency (E) of each gene was determined from the slope of the log template concentration (x-axis) - Ct value (y-axis) line, using the formula E = 10^−1/slope^ − 1^51^. The expression levels of *L-ACY-1* and the three reference genes in the larval midguts from different feeding treatments of the two *Helicoverpa* species were calculated with their mean Ct and amplification efficiency (*E*) (Equation 1). The expression levels of *H. armigera* and *H. assulta L-ACY-1* were further normalized with the geometric mean of the expression of the three reference genes (*RPL-13, RPL-32* and *β-Tubulin*) (Equation 2).

1 $${\rm{Expression}}\,{\rm{level}}={(1+{E}_{{\rm{gene}}})}^{-C{t}_{{\rm{gene}}}}$$

Normalized expression level of target gene (*L-ACY-1*).

2 $$=\,\frac{{(1+{E}_{{\rm{target}}{\rm{gene}}})}^{-C{{\rm{t}}}_{{\rm{targetgene}}}}}{\sqrt[3]{{(1+{E}_{RPL13})}^{-C{t}_{RPL13}}\times {(1+{E}_{RPL32})}^{-C{t}_{RPL32}}\times {(1+{E}_{\beta -TUB})}^{-C{{\rm{t}}}_{\beta -TUB}}}}$$

### Statistical analysis

Two-way ANOVA was performed to examine the impacts of species, feeding treatment and species × feeding treatment interaction on the expressions of *L-ACY-1*. Independent t-tests were conducted to test the significance of differences in the expression of *L-ACY-1* for all treatments between the two *Helicoverpa* species. Tukey’s HSD tests were performed to test the significance of differences in the expression levels of *L-ACY-1* among host plants, diets with different P:C ratio or plant allelochemicals within each species. All of the statistical tests were performed by SPSS version 19.0 software.
